# Exposure to Perfluorooctane Sulfonate In Utero Reduces Testosterone Production in Rat Fetal Leydig Cells

**DOI:** 10.1371/journal.pone.0078888

**Published:** 2014-01-14

**Authors:** Binghai Zhao, Li Li, Jieting Liu, Hongzhi Li, Chunlei Zhang, Pengfei Han, Yufei Zhang, Xiaohuan Yuan, Ren Shan Ge, Yanhui Chu

**Affiliations:** 1 Heilongjiang Key Laboratory of Anti-fibrosis Biotherapy, Heilongjiang, P. R. China; 2 Hong Qi Hospital, Mudanjiang Medical University, Heilongjiang, P. R. China; 3 The 2nd Affiliated Hospital of Wenzhou Medical University, Wenzhou, Zhejiang, China; Clermont Université, France

## Abstract

**Background:**

Perfluorooctane sulfonate (PFOS) is a synthetic material that has been widely used in industrial applications for decades. Exposure to PFOS has been associated with decreased adult testosterone level, and Leydig cell impairment during the time of adulthood. However, little is known about PFOS effects *in utero* on fetal Leydig cells (FLC).

**Methods and Results:**

The present study investigated effects of PFOS on FLC function. Pregnant Sprague Dawley female rats received vehicle (0.05% Tween20) or PFOS (5, 20 mg/kg) by oral gavage from gestational day (GD) 11–19. At GD20, testosterone (T) production, FLC numbers and ultrastructure, testicular gene and protein expression levels were examined. The results indicate that exposures to PFOS have affected FLC function as evidenced by decreased T production, impaired FLC, reduced FLC number, and decreased steroidogenic capacity and cholesterol level *in utero*.

**Conclusion:**

The present study shows that PFOS is an endocrine disruptor of male reproductive system as it causes reduction of T production and impairment of rat fetal Leydig cells.

## Introduction

Testicular dysgenesis syndrome (TDS) refers to a spectrum of reproductive disorders that originate in male fetal life [1]. TDS includes cryptorchidism (undescended testes) and hypospadias (abnormal formation of the urethral meatus) in newborn boys and testicular cancer and reduced fertility in adult males. Environmental endocrine disruptors (EEDs) are suspected contributors to TDS, as exposure to these substances has been related to reproductive tract anomalies, including cryptorchidism and hypospadias [2]–[4]. More than 200 chemicals meet the criteria for classification as EEDs, including compounds such as plasticizers, pesticides, natural plant metabolites, detergents and metals [5]. One class of EEDs is perfluoroalkylated substances (PFASs), among them perfluorooctane sulfonate (PFOS) is one of the synthetic materials that have been used in industries for decades as surface anti-fouling agents for textiles, leather products, furniture, carpets, and other materials [6]. PFOS has been found to accumulate in mammals, including humans [7]. PFOS concentration in human blood, ranges 14 to 59 ng/ml, around the world [8]–[12], and it was eliminated in serum for 5.4 years [8]. PFOS can be transferred to newborn across the placenta barrier [13]–[17]. Furthermore, PFAS levels in the range of 5 to 9.92 ng/ml were detected in the umbilical cord blood in Taiwan [18]. Among the PFAS, the transplacental crossing efficiency is the highest for perfluorooctanoic acid (PFOA) followed by perfluorohexane sulfonate (PFHxS) and is the lowest for PFOS [15] [16]. Controversy exists as to whether PFOS, at the level found in the environment, is harmful to humans, as most studies have been conducted in rodents following high doses. However, *Olsen etal* reported that PFOS levels can be high in serum of general population. Levels of more than 1500 ng/ml have been observed in American blood donors [6], [19]. Furthermore, occupational exposure can lead to serum levels greater than 12.8 µg/ml in fluorochemical manufacturing workers [20]. Thus, PFOS has been the focus of ongoing toxicology studies including those by national environmental management agencies [21].

Rats after exposure to PFOS or PFOA lead to adult Leydig cell (ALC) hyperplasia and eventually Leydig cell adenomas, and decreased testosterone (T) production [6], [22]–[24]. In our previous *in vitro* study, we showed that in ALCs perfluoroalkylated substances strongly inhibit 3beta-hydrosteroid dehydrogenase (3β-HSD) and 17beta-hydrosteroid dehydrogenase 3 (17β-HSD3), two enzymes associated with T production. Such inhibition was dependant on specific type of PFASs, PFOS(K)>PFOA>PFHxSK (potassium PFHxS) = PFBSK (potassium perfluorobutane sulfonate) [25]. However, the effects of PFOS on fetal Leydig cell (FLCs) have not been tested carefully in either human and or in rodents.

Two different populations of Leydig cells are recognized in the testis of rodents: Fetal Leydig cells and adult Leydig cells. These two types of cells exhibit distinctive topography, structure, ultrastructure, life span, capacity for androgen production, and mechanism of regulation by pituitary gonadotropins and growth factors [26]–[28]. FLCs secrete high levels of T that promote development of the penis and sex accessory glands, as well as insulin-like growth factor 3 (INSL3) necessary for scrotal descent of the testis. FLCs start to appear in the mesenchyme of the developing testis immediately following the formation of testicular cords (gestational day, GD, 11∼14.5 in rats) and reach their peak numbers around birth and then gradually involute after postnatal day 7 [29]. The goal of the present study was to examine the dose-dependent effects of PFOS on the prenatal production of T by FLCs. We postulated that FLC responds differently to PFOS compared to ALC. Given the essential role of fetal T production, our objective was to determine whether and how PFOS treatment in utero perturbs these cells and subsequently steroidogenesis function in the fetus.

## Materials and Methods

### Animals and treatment

Pregnant Sprague-Dawley rats (Vital River Laboratories, Beijing, China) were individually housed (23±2°C, relative humidity 55±5%), in a 12 hr light-dark cycle environment. The animals were housed in IVC cages (one rat per cage) on soft chip bedding and provided pellet chow (Vital River Laboratories). This study was approved by the Mudanjiang Medical University Ethics Committee, and all procedures were performed in accordance with the policies. The investigation conformed to the procedures described in the Guide for the Care and Use of Laboratory Animals published by the United States National Institutes of Health (NIH Publication No. 85-23, revised 1996). The pregnant dams were gavaged with PFOS at 0 (control), 5, or 20 mg/kg BW (PFOS kindly provided by Dr. Ren-Shan Ge of Wenzhou Medical University) in 0.05% (w/v) Tween 20 vehicle daily between GD11 and GD19 (*n* = 4 for each group). Pregnant dams were asphyxiated with CO_2_ at GD20.5, and the fetuses were then removed by caesarean section. Following measurement of the pups body weight and length, and AGD (anogenital distance) of the male pup of each pregnant dam, the pups were put into the airtight IVC cages and were sacrificed by asphyxiation with CO2. The fetal testes were immediately removed, frozen in liquid nitrogen and stored at −80°C. Each dam was weighed both at the start and at the end of the experimental period.

### Real-time polymerase chain reaction (PCR)

Total RNA was extracted from fetal testes using TRIzol (Invitrogen, Carlsbad, CA) according to the manufacturer's instructions. First-strand cDNA was synthesized from 2 µg of total RNA using Moloney murine leukemia virus reverse transcriptase and a Random Primers kit (Promega, Madison, WI). The ribosomal protein S16 mRNA level served as the internal control [30]. The primers used for PCR were provided in Table S2.

### Testicular T Analysis

The T content in testis was measured using an ELISA kit (Cusabio Biotech Co., Ltd., China). The tissues were rinsed with 0.01 M PBS, homogenized in 150 µl of 0.01 M PBS and stored overnight at −20°C. Two freeze-thaw cycles were performed and the material was centrifuged for 5 minutes at 5000 g in 4°C. Supernatants were collected and assayed according to the manufacturer's instructions.

### Other Assays

Mother serum and pup liver cholesterol levels were measured using an auto analyzer (AU 640, Olympus, Japan). Pup testis cholesterol, 3β-HSD, 17α-hydroxylase/20-lyase (P450c17), luteinizing hormone receptor (Lhcgr), scavenger receptor class B member 1 (SCARB1), clusterin (TRMP2), and progesterone levels were measured by specific ELISA kits (CUSABIO BIOTECH CO., LTD., China). The assays were preformed following collection of supernatants from tissue homogenates as described above.

### Western-blotting

Testicular tissues were homogenized in ice-cold 0.01 M phosphate-buffered saline containing 0.25 M sucrose. Total protein was quantified using the reagent of a BCA assay kit (Galen Biopharm,Bei Jing, China). To confirm the presence of B-cell lymphoma 2 (BCL2) and steroidogenic acute regulatory protein (STAR), 20 µg of total protein was fractionated using an SDS–PAGE gel (10% w/v acrylamide) and then blotted onto a polyvinylidene fluoride membrane (Bio-Rad, Hercules, CA). Nonspecific sites were blocked with nonfat milk powder (5% w/v) in PBST. The membrane was then incubated with rabbit monoclonal anti-BCL2 (Cell Signaling, China), mouse monoclonal anti-STAR (Abcam, HongKong, CA), and then with horseradish peroxidase–conjugated goat anti-rabbit, or anti-mouse secondary IgG (Abcam, HongKong, CA). Blots stripped and re-incubated with rabbit polyclonal to β-Actin (Abcam, HongKong, CA) served as the internal control.

### Enzyme Activity Assay

Tissues were homogenized in 0.01 M phosphate-buffered saline (PBS) containing 0.25 M sucrose. Homogenates were centrifuged at 700× *g* for 30 min at 4°C. The protein concentrations were then measured. Activities for testosterone biosynthetic enzymes activities 3β-HSD1 and P450C17 were determined by thin layer chromatography (TLC) as previously described [31]. The reaction mixtures (total volume of 250 µl) containing 25–160 µg protein, 0.2 mM cofactors (NAD+ for 3β-HSD1, NADPH for P450c17), and 10–1000 nM steroid substrates (radiolabeled + cold substrates) were incubated in shaking water bath at 37°C for 1 to 3 hr. The substrates were pregnenolone for 3β-HSD1 and progesterone for P450c17. The preliminary experiment was conducted to determine the linear reaction curve using varied concentrations of proteins at different time periods. Total steroids were extracted from reaction mixture with 1 ml of ice-cold ether, and the organic layer was evaporated under nitrogen gas. The extracted steroids were re-suspended in 70 µl ether and then spotted on thin layer plates (Baker-flex, Phillipsburg, NJ). They were separated chromatographically in chloroform: methanol (97∶3, v/v) for 3β-HSD1, as well as chloroform-ether (7∶1, v/v) for P450c17. The radioactivity was measured with a scanning radiometer (System 200/AC3000, Bioscan, Inc., Washington DC). The conversions of steroids to products were calculated as a percentage of the total radioactivity in the product. All assays were performed in triplicate.

### 3β-HSD staining of testis sections and the enumeration of FLC

Frozen testes were sectioned (6 µm) using a cryostat. FLCs were detected by histochemical staining for 3β-HSD activity with 0.4 mm etiocholanolone as the steroid substrate as described [32]. FLCs were identified by the staining of blue color in the cytosol. To enumerate FLC numbers, testicular tissues were sampled according to the Fractionator technique. The total number of FLCs per testis was calculated by multiplying the number of FLCs counted in a known fraction of the testis by the inverse of the sampling probability, and average FLC numbers per testis per treatment group were determined.

### Terminal deoxynucleotidyl transferase dUTP nick-end labeling staining

Apoptosis of testicular cells was determined by staining cryosections of rat testis (6 µm) using the In situ Cell Death Detection Kit (terminal deoxynucleotidyl transferase dUTP nick-end labeling [TUNEL] fluorescence FITC kit, Roche, Indianapolis, IN) according to the manufacturer's instructions. Following TUNEL staining, the sections were stained with a DAPI (BEYOTIME CO., China) to highlight the nuclei. Fluorescence staining was viewed by laser scanning confocal microscopy (FV300, Olympus, Japan). TUNEL-positive cells were counted in 3 fields (×400 magnification) of each cryosection.

### Ultrastructure observation

Testicular testis tissues were fixed in 2.5% glutaraldehyde in 0.1 M phosphate buffer (pH 7.4) at 4°C for 24 h, the samples were washed with phosphate buffer (0.1 M, pH 7.4) for 12 h and postfixed for 20 min in 1% OsO4 in 0.1 M phosphate buffer (pH 7.4). The samples were then washed with phosphate buffer (0.1 M, pH 7.4) for 30 min, dehydrated and embedded in Epon. Thin sections (50 nm) were placed on copper grids and stained in 2% uranyl acetate solution and 1% solution of lead citrate for 30 min. A JEM -1010 transmission electron microscope was used to visualize the ultrastructure. From each specimen 10 randomly micrographs were selected and analyzed using Image ProPlus software (Image-Pro Plus, Media Cybernetics,Shang Hai,China).

### Statistical analysis

Data are expressed as the mean ± standard error of the mean, and were analyzed using the one-way analysis of variance using Prism (version 4, GraphPad Software Inc., San Diego, CA) and Dunnett's comparison. The values were considered significantly different if p<0.05.

## Results

### General reproductive toxicology

The pregnant rats when gavaged with PFOS had no effect the number of pups and sex ratio per dam (Table 1). However, both BWs of the dams and neonates were significantly reduced (*p*<0.001) when the dams had received PFOS at 20 mg/kg BW, as were body lengths of the pups and testicular weights of male pups (*p*<0.001; *p*<0.05; respectively; Table 1). As AGD is a function of androgen action, we examined whether PFOS treatment caused a reduction in T synthesis by the fetal testis. In the present study, the AGDs of the male pups were significantly reduced (*p*<0.001) at a dose of 20 mg/kg PFOS (Table 1).

**Table 1:** General toxic parameters before and after exposure to PFOS.

	PFOS, mg/kg per day		
parameters	0	5	20
Dams			
Body weight before, g	254±9(4)	270±6(4)	259±11(4)
Body weight after, g	329±10(4)	343±6(4)	212±12(4)*** ¶
Pup male,%	23/45	31/57	19/36
Male pups			
Body weight, g	2.73±0.04(19)	2.52±0.03(19)** ¶	2.03±0.07(19)*** ¶
Body length, cm	3.24±0.03(19)	3.12±0.02(19)	2.61±0.08(19)*** ¶
AGD, mm	2.93±0.10(19)	2.78±0.07(19)	2.60±0.06(19)** ¶
TW, mg	6.26±0.35(19)	5.73±0.29(19)	4.93±0.33(19)* ¶

Dams of SD rats were gavaged with PFOS from GD11 to GD19. Parameters were measured at GD20.5. Values are mean ± SEM, (n) for dam's data.

,*P<0.05*,

,*P<0.01*,

,*P<0.001.*

One-way ANOVA with Dunnett's Multiple Comparison Test vs. control, significantly higher than control values.

### Testicular T production

**Figure 1:**
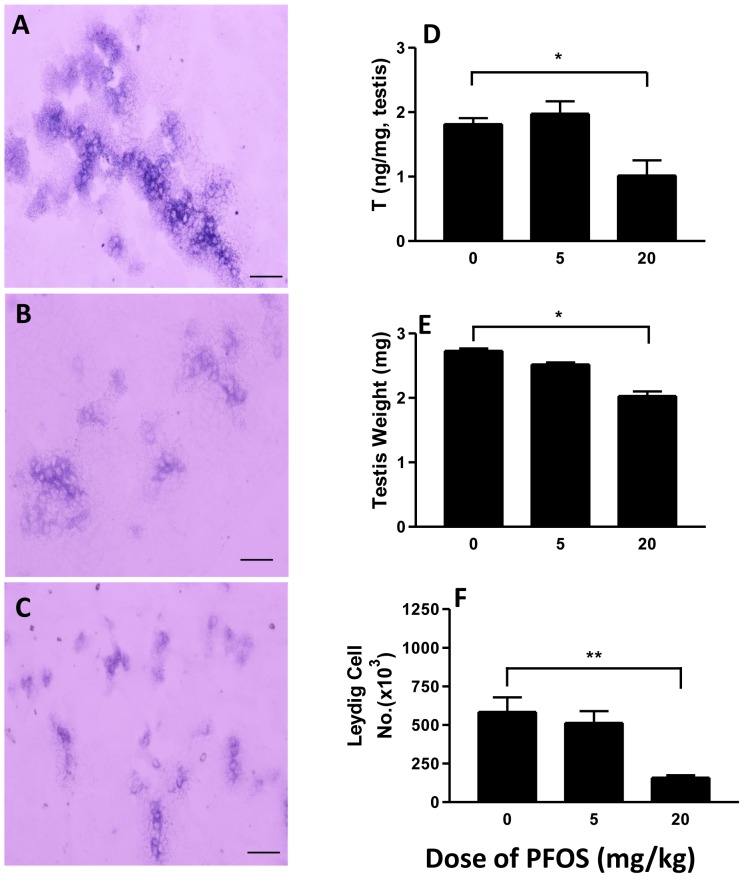
Effects of in utero PFOS exposure on (A–C) 3β-HSD for Leydig cells and testicular T (D) levels (n = 8), testicular weights (n = 19), and Leydig cell number (F). Pregnant dams were gavaged with 0 (control), 5, 20 mg/kg PFOS from GD11 to GD19. Measurements were made in male pups on GD20.5. Data are represented as mean ± SEM. A, B and C represent histochemical staining of 3βHSD at the doses of 0 (control), 5, 20 mg/kg PFOS. *^,^ ** indicates a significant difference compared to control (PFOS 0 mg/kg) at *P<0.05,P<0.01*, respectively. Bar = 50 µm.

Intratesticular T concentrations of male pups at GD20.5 were measured to assess the steroidogenic function of the FLCs. Relative to controls, T values were approximately 50% lower in the 20 mg/kg PFOS exposure group (Fig. 1D). No significant changes were observed in other groups.

### Testicular cell gene expression

**Figure 2:**
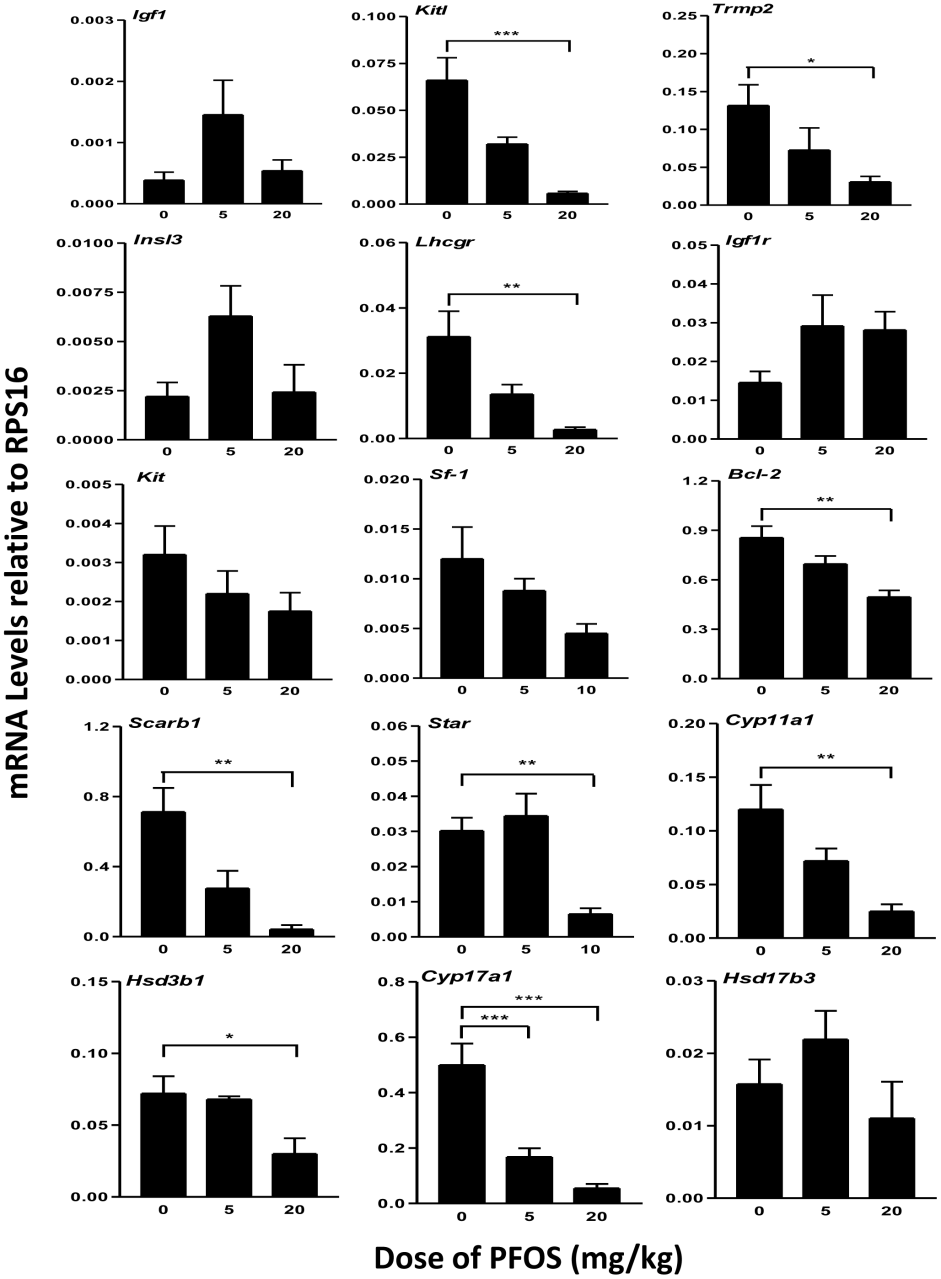
Real time PCR analysis of mRNAs in testis from GD20.5 after in utero PFOS exposures. Pregnant dams were gavaged with 0, 5, or 20/kg PFOS from GD11 to GD19. Gene symbols and the function of their products are reported in Table S1, S2. Data are presented as mean ± SEM (n = 4). *^,^*** indicates significant difference compared to control (PFOS 0 mg/kg) was shown at, *P<0.05*, *P<0.001*, respectively.

**Figure 3:**
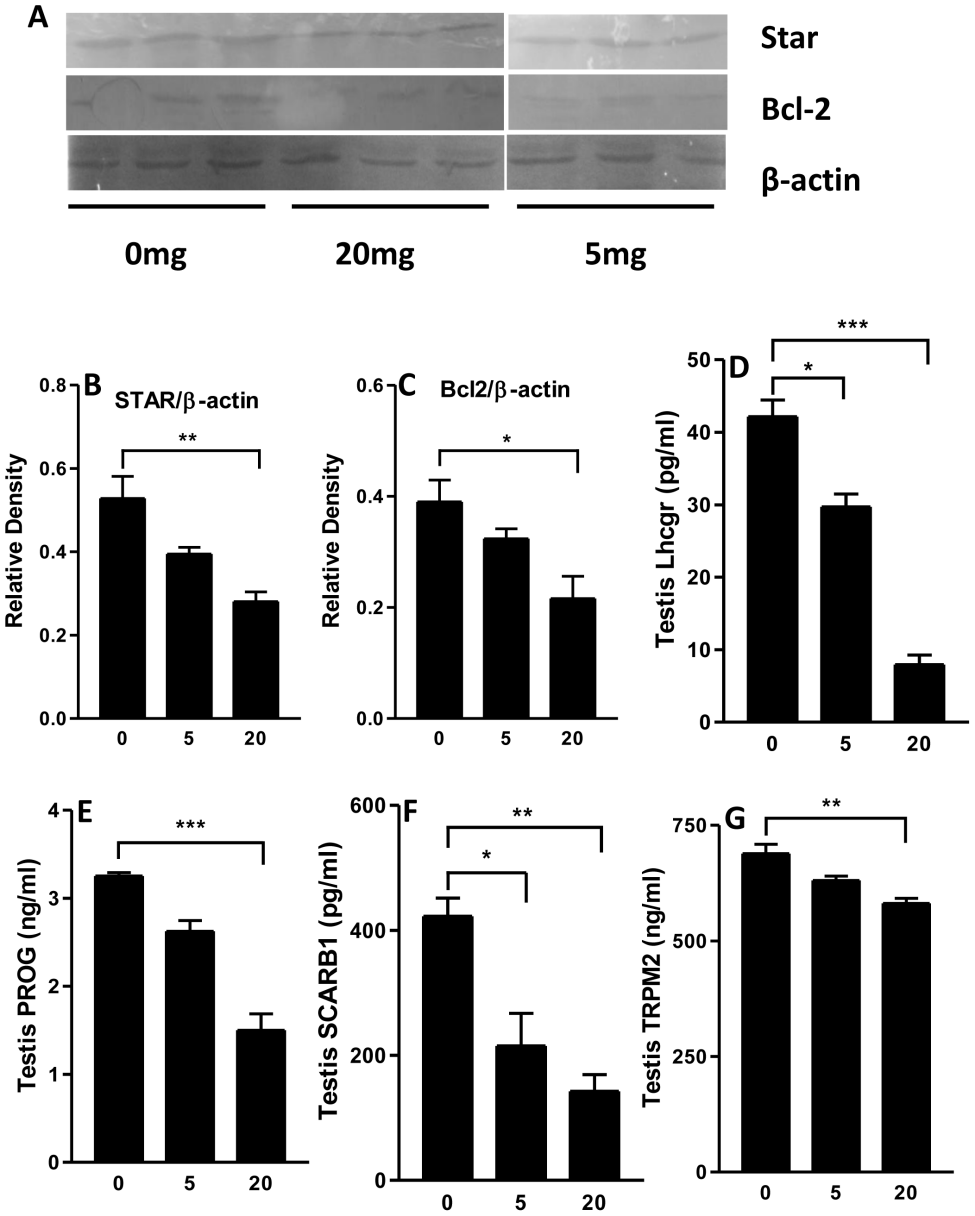
A–C: Western blots for BCL-2 and STAR from total pup testicular protein, β-actin served as the control. The data are shown as mean ± SEM (*n* = 3), *, *p*<0.05; ***, *p*<0.001, with respect to the control group levels. D–G: ELISA for Lhcgr, PROG (progesterone), SCARB1, TRMP2. Data are presented as mean ± SEM (n = 5). *^,^**^,^***indicates significant difference compared to control (PFOS 0 mg/kg) was shown at, *P<0.05*, *P<0.01*, *P<0.001*, respectively.

A series of mRNA transcripts were selected to examine cell type-specific testicular function following prenatal exposure to PFOS. As shown in Figure 2, the markers include genes encoding growth factors (*Igf1, Kitl*), their receptors (*Igf1r, Kit*), cholesterol transporters (*Scarb1, Star*), steroidogenic enzymes (*Cyp11a1, Cyp17a, Hsd17b3*), and junction protein (*Trmp2*). Overall transcript levels of 17 testicular mRNAs were examined by real-time PCR. The gene names, symbols, and functions are listed in Table S1. Among them, *Kitl*, *Scarb1* and *Star* mRNA levels were significantly decreased in the 20 mg/kg PFOS group. No change was observed in the expression levels of *Kit* and *Igf1r*. The protein levels of STAR and SCARB1 were also decreased (Fig. 3B,F). The steroidogenic enzyme genes, *Cyp11a1* (20 mg/kg), *Cyp17a* (5, 20 mg/kg), and *Hsd3b1* (20 mg/kg) showed reduced levels of expression in response to PFOS. The apoptosis related protein and junction protein gene , *Bcl2* and *Trmp2* mRNA and protein levels in 20 mg/kg were decreased when compared to controls (Fig. 3C,G). Although FLC differentiation is LH-independent, LH receptor (*Lhcgr*) drives fetal T secretion [33]. In this study, the *Lhcgr* mRNA and protein levels were also significantly decreased in 20 mg/kg dose compared to control (Fig. 3D). These changes are consistent with reduced intratesticular T levels (Fig. 1). Interestingly, *Insl3*, the protein involved in testis descent, was unchanged in the 20 mg/kg PFOS group.

### T biosynthetic enzyme protein levels

**Figure 4:**
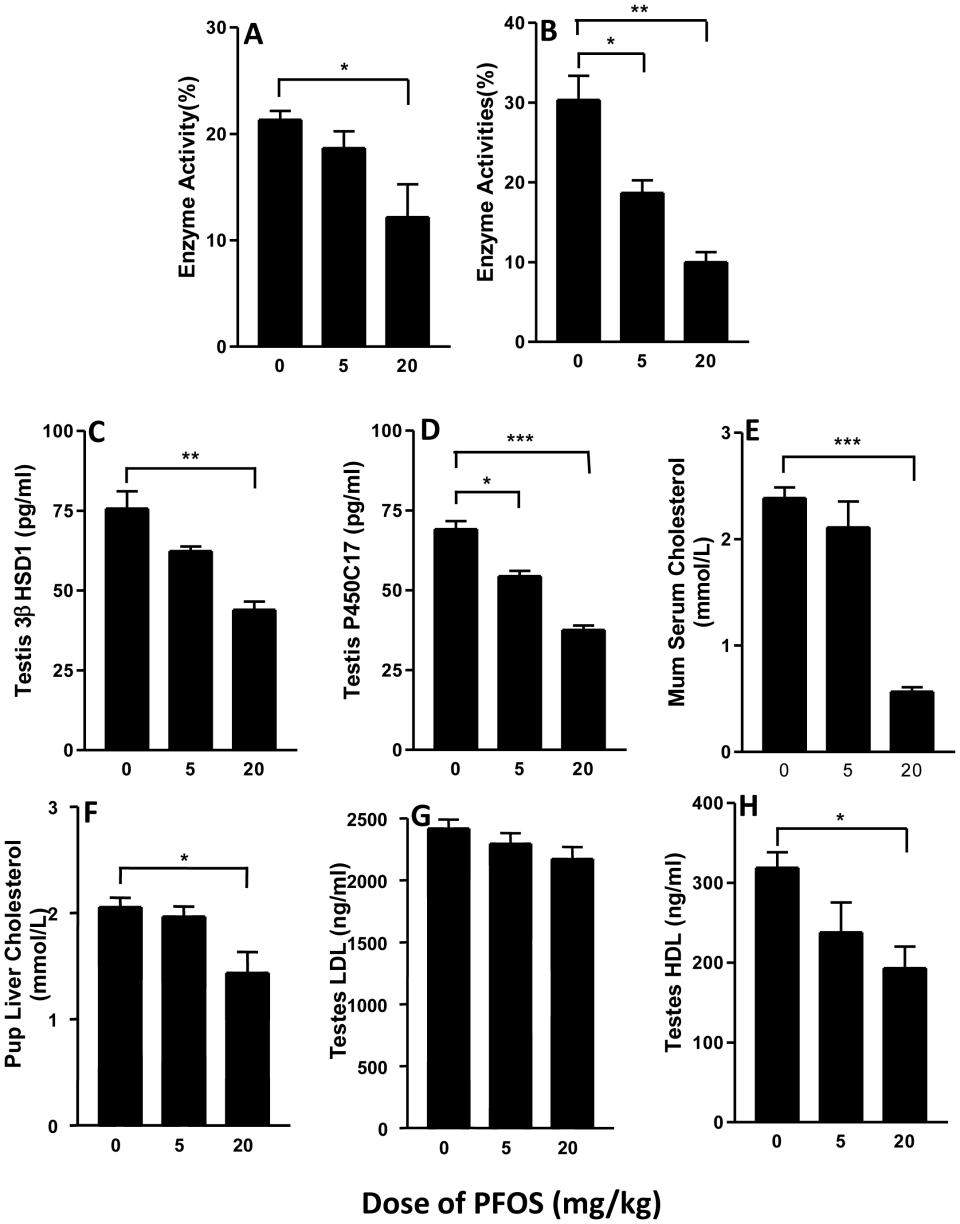
A–D: Enzyme activities and protein levels of 3β-HSD1 (A,C), P450c17 (B,D), in fetal testis from GD20.5 following in utero PFOS exposures. Pregnant dams were gavaged with 0, 5, or 20 mg/kg PFOS from GD11 to GD19. The enzyme activities were measured as described in Materials and Methods. Data are presented as mean ± SEM (n = 5∼6). E–H: quantification of mother serum (n = 3) , male pup liver cholesterol (n = 8) and testicular LDL or HDL (n = 8). *^,^**^,^*** indicates significant difference compared to control (PFOS 0 mg/kg) was shown at, *P<0.05*, *P<0.01*, *P<0.001*, respectively.

T biosynthetic enzyme protein levels were also evaluated. 3β-HSD and P450c17 enzyme activity and protein levels were reduced at high PFOS dose (Fig. 4 A–D). These results suggest that reduced levels of 3β-HSD and P450c17 might be integrally involved in PFOS-mediated inhibition of T production at the higher PFOS dose.

### Cholesterol levels

Cholesterol is the source of testosterone biosynthesis. There are mainly three potential sources, which could contribute to the putative “cholesterol pool” needed for T production: (a) stored cholseteryl esters (lipid droplets), (b) exogenous lipoprotein-supplied cholesterol (low-density lipoprotein, LDL), (c) plasma membrane-derived cholesterol (high-density lipoprotein, HDL) [34]. Among these, cholesterol-rich plasma lipoproteins are often the most utilized source of cholesterol for T synthesis. To determine if the decreased T production was caused by cholesterol insufficiency, we measured mother serum and pup liver and testis cholesterol levels. As shown in Figure 4, mother serum cholesterol levels was significantly decreased in the animals treated with a 20 mg/kg dose of PFOS compared to controls (*P<0.001*, Fig. 4E). Similarly the HDL levels of the male pup liver and testis (*P<0.05*, Fig. 4F,H) were also reduced. Thus the inadequate cholesterol is one of the reasons for T deficiency.

### Leydig cell number, apoptosis, ultrastructure and size

**Figure 5:**
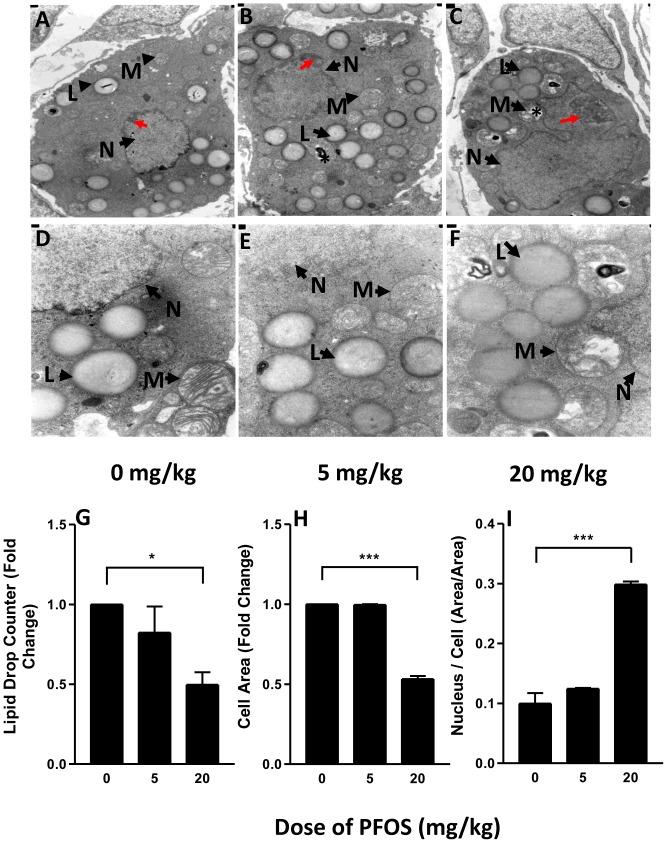
Ultrastructure of fetal Leydig cells (FLC) from male rats exposed to PFOS or vehicle from GD11 to GD19. L indicates lipid droplet; M indicates mitochondria; N indicates nucleus; red arrows indicate condensed chromatin; asterisk indicates swollen mitochondria. Quantification of ultrastructure of FLC from male rats exposed to PFOS or vehicle from GD11 to GD19. (A) the number of lipid droplets (n = 15), (B) FLC area (n = 15), (C) the ratio of nuclear area/cell area (n = 15). *,*** indicates significant difference compared to control (PFOS 0 mg/kg) was shown at, *P<0.05*, *P<0.001*, respectively.

**Figure 6:**
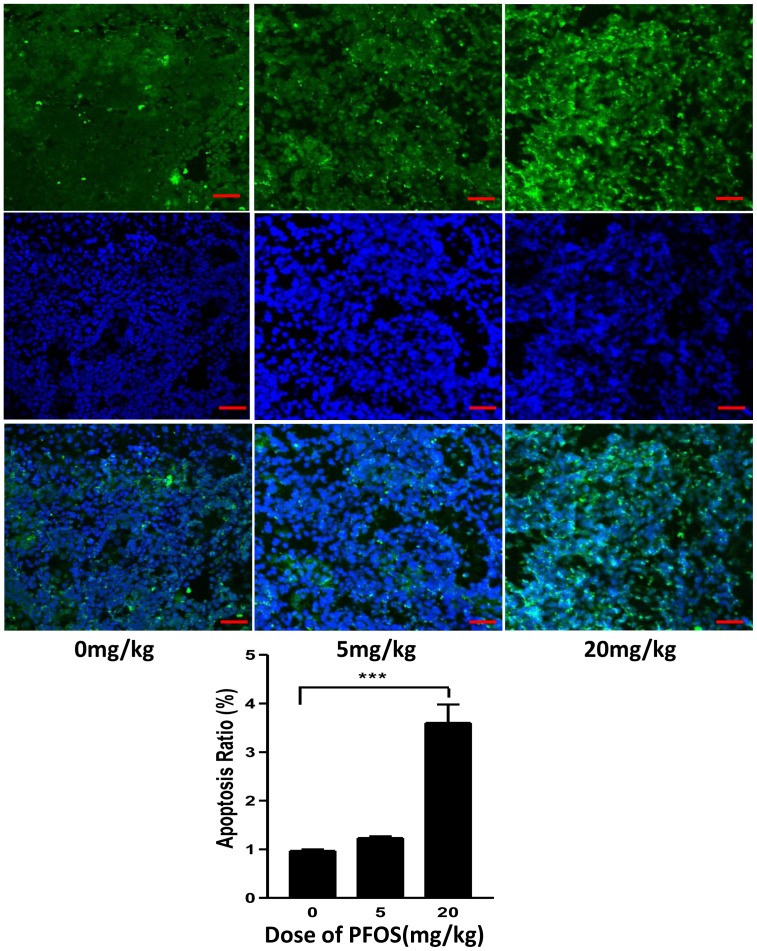
TUNEL staining of fetal testis from GD20.5 following in utero PFOS exposure. Pregnant dams were gavaged with 0, 5, or 20/kg PFOS from GD11 to GD19. Apoptosis rates are expressed as mean±SEM; (n = 5). ***P<0.001 vs Control. Bar = 50 µm.

FLCs are not uniformly distributed in the interstitial space of the testis but rather are found in discrete clusters [35]. FLCs exhibit some aspects of differentiated function, including 3βHSD activity [36]. In the present study, we use 3βHSD staining to determine FLC numbers. Compared to controls, the number of FLCs showed a significant reduction at a dose of 20 mg/kg PFOS. This is evident in micrographs (Fig. 1A, B, C, F). Such reduced FLC numbers respond to PFOS exposures of 20 mg/kg were associated with reduced intratesticular T concentrations and testicular weights (Fig. 1D, E). For vehicle-treated rats, FLCs in the testes showed normal ultrastructure with intact nuclei and varied numbers of lipid droplets and organelles (Fig. 5A, D). Although the ultrastructure in the FLCs seemed relatively undamaged in rats receiving 5 mg/kg PFOS, the nuclei were enlarged and chromatin was partly condensed (Fig. 5B, red arrow), with scattered swollen mitochondria, and with disintegrated cristae (Fig. 5E, asterisk). In rats treated with 20 mg/kg PFOS, the FLCs showed decreased number of lipid droplets and features of apoptosis including enlarged nuclei, condensed chromatin, and vacuolated mitochondria (Fig. 5C, F). Taken together, these observations indicate that features of apoptosis are common in FLC treated high dose of PFOS, which is consistent with the TUNEL staining and BCL-2 protein levels in testes of rats exposed to 20 mg/kg PFOS (Fig. 6).

## Discussion

Previous studies examining PFOS exposure to adulthood testis have focused mainly on ALC and sperm. Here we determined whether and how PFOS affect FLC, as well as disrupt T production, at the prenatal stage . It is widely accepted that at least two populations of Leydig cell, FLC and ALC, exist at different development stages in most mammalian testes. FLC derived T that is crucial for the development of the penis and sex accessory glands and both T and INSL3 are involved in testicular descent. In the present study, we found that T levels were significantly lower in high doses of PFOS relative to control, while INSL3 was unchanged. Such effects of PFOS were also previously shown *in vitro* experiments, using 10 or 100 µM PFOA, another perfluoroalkylated substance that is structurally related to PFOS [24]. Thus, interference with the development of FLCs may be a precipitating cause of TDS. TDS can occur as a result of abnormal Leydig cell function. Such notion is also supported by reduced testicular weights is associated with T levels in the present study. Overall the results suggest that PFOS may be a contributor to TDS.

Reduced AGDs are considered to be a reliable surrogate marker of decreased T levels [37]. In this study, PFOS exposure in utero caused decreased AGD as well as reduced T levels and testes weight, indicating PFOS may act as an anti-androgen, affecting FLC steroidogenesis. In keeping with this notion, significant down-regulation of components of the steroidogenic pathway, including cholesterol transporting protein *Scarb1* and *Star* and steroidogenic enzyme gene such as *Cyp11a1*, *Hsd3b1*, and *Cyp17a1* were observed following high dose of PFOS treatment in the present study. These results indicate a reduction of molecules involved in T synthesis through both LH-dependent and independent mechanisms following PFOS exposure. Steroidogenic factor (SF-1), a product of the sex determining region on the Y chromosome, SRY, directs fetal Leydig stem cells toward lineage-specific development and steroidogenic competence [38]. SF-1 also stimulates expression of the cytochrome P450 enzymes of steroid synthesis. Conditional knockout of SF-1 in the Leydig cells lead to undetectable levels of P450scc [39]. In the present study, although SF-1 mRNA levels showed no significant difference in 5 and 20 mg/kg doses, a decreased tendency towards reduction was found in 20 mg/kg dose. Furthermore, testis cells including FLCs showed increased apoptosis rate and ultrastructural alteration, indicating impaired FLC at the doses of 5 and 20 mg/kg PFOS. These cells exhibited small cell size, little lipid droplet, enlarged nuclei or vacuolated mitochondria, and condensed chromatin. Such alterations in FLC reflect that high dose PFOS induce apoptosis, in theory which alters mRNA levels of *Bcl2* and *Trpm2*. TRPM-2 (Sertoli cell marker), also known as clusterin, is believed to be associated with apoptosis as its expression is increased in the regressing prostate following androgen ablation [40]. In addition, TRPM-2 has been shown to inhibit apoptosis and enhance survival of cells in culture [41]. Decreased *Bcl2* expression is also related to cell survival in the testes [42]. The alteration of *Trpm2* and *Bcl2* mRNA and TRMP2 and BCL-2 protein levels are consistent with increased apoptosis shown by TUNEL staining. Together, these results suggest that regions of FLCs damage are, in part, due to enhancing of apoptosis and reduced cell survival.

Mitochondria play key roles in the production of T, through important proteins - StAR and P450scc. StAR protein mediates the rate-limiting step in steroidogenesis, i.e. the transportation of cholesterol from the outer mitochondrial membrane to the inner mitochondrial membrane [43]. P450scc encoded by gene *Cyp11a1* catalyzes the conversion of cholesterol to pregnenolone, the first and rate-limiting step in the biosynthesis of testosterone. In this study, FLC mitochondria were found to be vacuolated and the cristae disintegrated, in parallel with decreased mRNA levels of StAR and P450scc.

In summary, the present study showed that PFOS is an endocrine disruptor of male reproduction system through reduction of T production and impairment of rat FLCs.

## Supporting Information

Table S1 **Leydig and Sertoli cell-related genes (16 genes).**
(DOCX)

Table S2 **Primers for Leydig and Sertoli cell-related genes (16 genes).**
(DOCX)
