# Supplementary material for: Exposure to Perfluorooctane Sulfonate In Utero Reduces Testosterone Production in Rat Fetal Leydig Cells
Source: PLoS One. 2014 Jan 14;9(1):e78888. doi: 10.1371/journal.pone.0078888 (PMC3891643; doi:10.1371/journal.pone.0078888)
Supplement: Table S1 — Leydig and Sertoli cell-related genes (16 genes). (DOCX) [file pone.0078888.s001.docx]

Table S1. Leydig and Sertoli cell-related genes (16 genes).

| Gene full name | Gene symbol | FLC-related effect |
| --- | --- | --- |
| Insulin like growth factor 1 | *Igf1* | Growth factors involing in Leydig development |
| Kit ligand | *Kitl* |  |
| Insulin like growth factor 3 | *Insl3* | Induction of testis decent |
| Luteinizing hormone receptor | *Lhcgr* | involving in Leydig cell development |
| Insulin-like growth factor 1 receptor | *Igf1r* |  |
| c-kit | *Kit* |  |
| Scavenger receptor class B type I | *Scarb1* | Cholesterol transport |
| Steroidogenic acute regulatory protein | *Star* |  |
| Steroidogenic factor 1(*Sf-1*) | *Nrd5a1* |  |
| B cell lymphoma/lewkmia-2 | *Bcl-2* | Related cell apoptosis |
| P450 Side-chain Cleavage Enzyme | *Cyp11a1* | Steroidogenesis |
| 3β-hydroxysteroid dehydrogenase 1 | *Hsd3b1* |  |
| P450 17α hydroxylase | *Cyp17a* |  |
| 17β-hydroxysteroid dehydrogenase 3 | *Hsd17b3* |  |
| Clusterin | *Trmp2* | Junction protein |
| Ribosomal protein 16 | *Rps16* | Internal control protein |
