# Supplementary material for: Exposure to Perfluorooctane Sulfonate In Utero Reduces Testosterone Production in Rat Fetal Leydig Cells
Source: PLoS One. 2014 Jan 14;9(1):e78888. doi: 10.1371/journal.pone.0078888 (PMC3891643; doi:10.1371/journal.pone.0078888)
Supplement: Table S2 — Primers for Leydig and Sertoli cell-related genes (16 genes). (DOCX) [file pone.0078888.s002.docx]

Table S2. Primers for Leydig and Sertoli cell-related genes (16 genes)

| Gene symbol | GeneBank accession no. | Forward primer | Reverse primer | Size,bp |
| --- | --- | --- | --- | --- |
| *Igf1* | NM_178866 | 5’ACTCTGCTTGCTCACCTTTACC3’ | 5’TCATCCACAATGCCCGTC3’ | 174 |
| *Kitl* | NM_021843 | 5’TGATAACCCTCAACTATGTCGC3’ | 5’GGTCATCCACTATTTTCCCAAG3’ | 176 |
| *Insl3* | NM_053680 | 5’GTGGCTGGAGCAACGACA3’ | 5’AGAAGCCTGGTGAGGAAGC3’ | 102 |
| *Lhcgr* | NM_012978 | 5’CTGCGCTGTCCTGGCC3’ | 5’CGACCTCATTAAGTCCCCTGAA3’ | 102 |
| *Igf1r* | L29232 | 5’CTGTGTTCTTCTATGTCCC3’ | 5’CGAGCTCCCGGTTCATGG3’ | 260 |
| *Kit* | D12524 | 5’ATCCAGCCCCACACCCTGTT3’ | 5’TGTAGGCAAGAACCATCACAAT3’ | 90 |
| *Scarb1* | NM_031541 | 5’ATGGTACTGCCGGGCAGAT3’ | 5’CGAACACCCTTGATTCCTGGTA3’ | 117 |
| *Star* | NM_031558 | 5’CCCAAATGTCAAGGAAATCA3’ | 5’AGGCATCTCCCCAAAGTG3’ | 187 |
| *Nr5a1* | NM_053344 | 5’CAGAGCTGCAAAATCGACAA3’ | 5’CCCGAATCTGTGCTTTCTTC3’ | 186 |
| *Bcl-2* | NM_016993 | 5'-AGCGTCAACAGGGAGATGTC-3' | 5'-TATGCACCCAGAGTGATGCA-3' | 224 |
| *Cyp11a1* | NM_017286 | 5’AAGTATCCGTGATGTGGG3’ | 5’TCATACAGTGTCGCCTTTTCT3’ | 126 |
| *Hsd3b1* | NM_017265 | 5’CCCTGCTCTACTGGCTTGC3’ | 5’TCTGCTTGGCTTCCTCCC3’ | 178 |
| *Cyp17a* | NM_012753 | 5’TGGCTTTCCTGGTGCACAATC3’ | 5’TGAAAGTTGGTGTTCGGCTGAAG3 | 90 |
| *Hsd17b3* | NM_054007 | 5’ TTTCTTCGGGAGTAGGGGTTC3’ | 5 TCATCGGCGGTCTTGGTCG3’ | 201 |
| *Trmp2* | M64723 | 5’CTGTTTGACTCTGACCCCATC3’ | 5’TTTCCTGCGGTATTCCTGTA3’ | 110 |
| *Rps16* | X17665 | 5’AAGTCTTCGGACGCAAGAAA3’ | 5’TGCCCAGAAGCAGAACAG3’ | 146 |
